# Therapeutic potential and mechanisms of rehabilitation interventions in preventing cancer metastasis

**DOI:** 10.3389/fonc.2026.1740636

**Published:** 2026-07-29

**Authors:** Xiaoyan Chen, Junfeng Zhang, Xiaoling Liu, Huijun Du

**Affiliations:** 1 School of Basic Medical Sciences, Hubei University of Chinese Medicine, Wuhan, China; 2 Taihe Hospital, Hubei University of Medicine, Shiyan, China; 3 Pediatric Department of Taihe Hospital, Hubei University of Medicine, Shiyan, China; 4 Department of Rehabilitation, Taihe Hospital, Hubei University of Medicine, Shiyan, China

**Keywords:** cancer metastasis, immune modulation, interdisciplinary teamwork, rehabilitation intervention, tumor microenvironment

## Abstract

Cancer, a significant global public health challenge, has had a persistent increase in both incidence and mortality rates, with metastasis accounting for nearly 90% of cancer-related deaths worldwide. Consequently, the efficient prevention of cancer metastasis has emerged as a vital subject in contemporary medical research and clinical practice at the global level. This narrative review summarizes preclinical and clinical evidence on the role of rehabilitation interventions in preventing cancer metastasis, and highlights the importance of interdisciplinary teamwork in accomplishing this objective. Rehabilitation intervention includes diverse treatment modalities, such as exercise therapy, physical agent modalities, occupational therapy, and psychological intervention. These approaches substantially enhance patients’ quality of life while concurrently impeding the proliferation and metastasis of cancer cells by regulating the immune system, diminishing chronic inflammation, affecting the tumor microenvironment (TME), and adjusting hormonal levels in the body. This paper does a thorough investigation of the impact of rehabilitation interventions on diverse cancer patients, elucidating the distinct functions of different rehabilitation measures across cancer types to assess their effectiveness in preventing metastasis. This article examines the probable molecular mechanisms of rehabilitation intervention, elucidating the pathways by which it mitigates the risk of metastasis through the regulation of the immune system and the TME. Future research should prioritize well-designed randomized controlled trials (RCTs) with metastatic-related outcomes, integrate biological measures such as immune profiling, cytokine levels, and gene expression, emphasize patient-centered rehabilitation programs tailored to immune/tumor biomarkers and cancer types, and ensure long-term follow-up of over one year to assess sustained efficacy and survivorship outcomes.

## 1 Introduction

**Figure 1:**
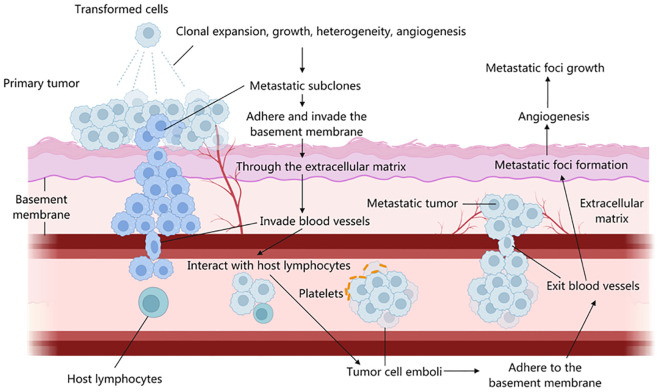
The main steps of the metastatic cascade. The process begins with clonal expansion and angiogenesis in the primary tumor. Metastatic subclones then invade the basement membrane and extracellular matrix to enter blood vessels. During circulation, tumor cells interact with platelets to form emboli, which helps them evade host lymphocytes. Finally, the surviving cells adhere to distant microvessels, exit the circulation, and establish metastatic foci fueled by secondary angiogenesis. Created by the authors using EdrawMax.

Cancer, a significant public health challenge globally, has shown a persistent increase in both incidence and fatality rates (1). According to the GLOBOCAN 2022 report, the number of new cancer cases worldwide has increased from approximately 10 million in 2000 to an estimated 20 million in 2022, reflecting a significant global rise (2). It not only presents a significant risk to human health but also has a substantial influence and considerable weight on global social and economic advancement. The economic burden is substantial, with global cancer care costs forecasted to exceed $500 billion by 2030, particularly driven by high-prevalence cancers such as breast, lung, and colorectal cancer (3). Cancer metastasis, the primary cause of cancer-related fatalities, has underscored the significance and immediacy of its prevention (4). Cancer metastasis is driven by a highly orchestrated and complex sequence of biological events collectively known as the metastatic cascade (5). This process initiates with the epithelial-mesenchymal transition (EMT), wherein primary tumor cells lose their cell-cell adhesion properties (such as the downregulation of E-cadherin) and acquire a migratory and invasive mesenchymal phenotype. Subsequently, these cells secrete matrix metalloproteinases (MMPs) to degrade the extracellular matrix and basement membrane, facilitating local invasion into the surrounding stroma. Following local invasion, tumor cells intravasate into the local microvasculature or lymphatic vessels. Once in the systemic circulation, circulating tumor cells (CTCs) face intense hemodynamic shear stress and immune surveillance, predominantly by natural killer (NK) cells. To survive, CTCs often associate with platelets to form tumor microemboli, effectively evading immune detection. Upon reaching distant microcapillary beds, these cells arrest, adhere to the endothelial lining, and undergo extravasation into the secondary organ parenchyma (6). Ultimately, the successful establishment of metastatic lesions requires the formation of a supportive pre-metastatic niche, metabolic adaptation, and secondary angiogenesis to sustain macroscopic growth (5) (**Figure 1**). This establishes a solid foundation for the role of rehabilitation interventions in avoiding cancer spread (7). Evidence suggests that exercise can modulate immune surveillance, reduce systemic inflammation, and alter the TME, while psychological interventions may lower chronic stress and associated pro-metastatic signaling (8). Rehabilitation intervention is essential in cancer management, incorporating exercise therapy, physical component treatment, and psychological intervention, aimed at enhancing patients’ quality of life (9). It also demonstrates potential in diminishing the danger of recurrence and metastasis. For example, a randomized controlled trial by Courneya et al. demonstrated that exercise interventions reduced recurrence and improved survival in breast cancer patients, while animal studies have shown that physical activity inhibits metastatic progression via immune-mediated pathways (10, 11). Due to the intricacy of cancer rehabilitation, an interdisciplinary approach is essential. This interdisciplinary intervention strategy enhances treatment outcomes and significantly improves patients’ quality of life and long-term prognosis (12). Research on the prevention of cancer metastasis is now advancing steadily, particularly in the area of rehabilitative intervention. Recent discoveries and technical advancements have revitalized active research in this domain, establishing a robust foundation for future inquiries and trajectories (13). A recent meta-analysis showed that regular physical activity is associated with a 27% reduction in cancer-specific mortality across multiple cancer (14). Moreover, structured rehabilitation programs have been linked to improved functional outcomes and survival rates in cancer patients (15).

While prior research has concentrated on enhancing the overall health of cancer patients with rehabilitation therapy, the distinct roles of these interventions in inhibiting the metastatic process have not been comprehensively summarized and examined (13). For example, systematic reviews by Cormie et al. and Mishra et al. have demonstrated that exercise-based rehabilitation improves physical function and overall survival in cancer survivors. However, few studies have specifically addressed how these interventions may influence metastatic progression (15, 16). This review seeks to furnish scientific direction for oncology rehabilitation practitioners, patients, and their families by comprehensively synthesizing research findings on several rehabilitation modalities, thereby enhancing their participation in the rehabilitation process. In this context, participation refers to both improved adherence to rehabilitation protocols and greater patient empowerment in shared decision-making. These practical recommendations may offer novel pathways for advancing cancer rehabilitation and stimulate development in allied fields such as nursing, exercise therapy, physiotherapy, psychology, nutrition, and social work. The practical applicability of the rehabilitation intervention techniques examined in this paper is substantial in medical practice. These strategies may help decrease the risk of metastasis in cancer patients. For instance, observational studies have reported that regular exercise is associated with a 20-30% reduction in cancer recurrence in certain types of cancer (14). In addition, rehabilitation interventions have been shown to improve survival rates, enhance quality of life, and reduce psychological distress in cancer patients. For example, a meta-analysis reported that exercise-based rehabilitation resulted in significant improvements in both physical and mental health-related quality of life scores (15), and was associated with lower rates of depression and anxiety (17).

## 2 Literature search and selection process

This article is presented as a narrative review rather than a systematic review or meta-analysis. While narrative reviews do not strictly mandate adherence to systematic protocols such as PRISMA, we have proactively incorporated several PRISMA-inspired elements to enhance methodological transparency and reproducibility. We conducted a comprehensive literature search across four major databases: PubMed, Embase, Web of Science, and Scopus, retrieving articles published between January 2005 and June 2026. The search strategy utilized the following keywords and Boolean operators: (“cancer metastasis” OR “tumor metastasis”) AND (“rehabilitation” OR “exercise therapy” OR “physical therapy” OR “psychological intervention”).

The inclusion criteria were defined as follows: (1) original research articles or reviews investigating rehabilitation interventions in the context of cancer metastasis; (2) studies involving *in vitro*, *in vivo*, or human subjects; and (3) publications written in English. Conversely, the exclusion criteria comprised: (1) non-peer-reviewed articles (e.g., preprints, conference abstracts); (2) studies not directly relevant to rehabilitation or metastasis; and (3) case reports or small case series with fewer than 10 subjects. To ensure objectivity, two independent reviewers initially screened all titles and abstracts for eligibility, followed by a rigorous full-text review for final selection. Any discrepancies between the reviewers were resolved through thorough discussion; in instances where a consensus could not be reached, a third independent reviewer was consulted for final adjudication.

## 3 The mechanism of rehabilitation intervention

### 3.1 Mitigate chronic inflammation and augment immunological activity

Rehabilitation interventions, particularly physical activity, confer both direct and indirect effects on cancer patients as they relate to metastasis prevention. The direct physiological effects include the robust modulation of immune function, regulation of hormonal levels, and comprehensive remodeling of the TME (18). Specifically, recent evidence reveals that regular exercise stimulates muscle fibers to secrete myokines and extracellular vesicles, which subsequently downregulate collagen composition in the extracellular matrix and promote the intra-tumoral infiltration and activation of cytotoxic CD8+ T cells and NK cells (19, 20). These exercise-induced metabolic and immunological shifts actively suppress chronic inflammation and create a less permissive microenvironment for metastatic colonization (21). In addition to these direct pathways, physical activity exerts a profound range of indirect benefits, such as alleviating psychological distress, significantly reducing cancer-related fatigue, and enhancing patients’ overall adherence to rigorous treatment regimens (22). Collectively, these multi-target neuroendocrine and immunological regulations synergize to improve the overall prognosis and quality of life in patients at risk for metastasis.

Rehabilitation intervention has a multifaceted and synergistic purpose in avoiding tumor spread by augmenting immune function and diminishing chronic inflammation (23). Moderate rehabilitation exercises, including brisk walking, swimming, and yoga, markedly restore patients’ cardiopulmonary function and physical fitness while simultaneously bolstering the body’s anti-cancer capabilities through intricate immune regulatory processes (24). Specifically, aquatic exercises such as swimming enhance lung capacity and respiratory muscle strength due to the hydrostatic pressure and constant resistance provided by water, thereby improving overall cardiopulmonary endurance (25). Furthermore, mind-body interventions like yoga not only increase musculoskeletal flexibility but also significantly reduce systemic stress and circulating cortisol levels through regulated breathing and mindfulness practices, which is crucial for downregulating pro-tumorigenic inflammatory pathways (26). Both activities have been shown to reduce cancer-related fatigue and improve emotional well-being in survivors, thus positively contributing to the cancer recovery process (27, 28). Aerobic exercise can markedly augment the activity of NK cells and macrophages, hence enhancing their capacity to identify and eradicate cancer cells (29). NK cells are a crucial element of the innate immune system, and their augmented functionality directly influences the immune evasion capabilities of tumor cells (30). Aerobic exercise enhances NK cell function, facilitating the release of cytotoxic granules like perforin and granzyme, which induce death in tumor cells (29). Macrophages, pivotal cells within the tumor microenvironment (31), can be converted from M2 pro-tumor phenotypes to M1 anti-tumor phenotypes via the modulation of aerobic exercise, thereby augmenting their phagocytic capacity against tumor cells and promoting the secretion of anti-inflammatory cytokines such as IL-12, which diminishes pro-inflammatory responses in the TME and curtails the proliferation and metastasis of tumor cells (18).

Consistent physical activity modulates the hypothalamic-pituitary-adrenal (HPA) axis, mitigates stress reactions, and diminishes the release of stress hormones, including cortisol and adrenaline (32). The overproduction of stress hormones facilitates tumor proliferation and metastasis, mostly by stimulating pro-inflammatory pathways, including NF-κB and STAT3 signaling, thereby enhancing the synthesis of pro-inflammatory cytokines like TNF-α and IL-6 (33). Exercise can diminish the levels of pro-inflammatory factors, hence mitigating the influence of chronic inflammation on tumor metastasis (34). Aerobic exercise simultaneously enhances the production of anti-inflammatory cytokines, such as IL-10, which regulates immunological equilibrium, suppresses chronic low-grade inflammation inside the TME, and diminishes the proliferative capacity of cancer cells (35). Recent preclinical and clinical studies have clarified that exercise induces systemic increases in IL-6 and IL-15, which are crucial for NK cell proliferation and cytotoxic activation via the STAT5 pathway. In breast cancer models, exercise-induced IL-15 signaling enhances NK cell expression of NKG2D receptors, facilitating recognition and lysis of metastatic tumor cells (11, 36). Similarly, in colorectal cancer, catecholamine-mediated β-adrenergic receptor activation mobilizes NK cells to the circulation and metastatic niches, where they exert anti-metastatic effects by secreting IFN-γ and downregulating tumor-associated MMPs (37).

**Figure 2:**
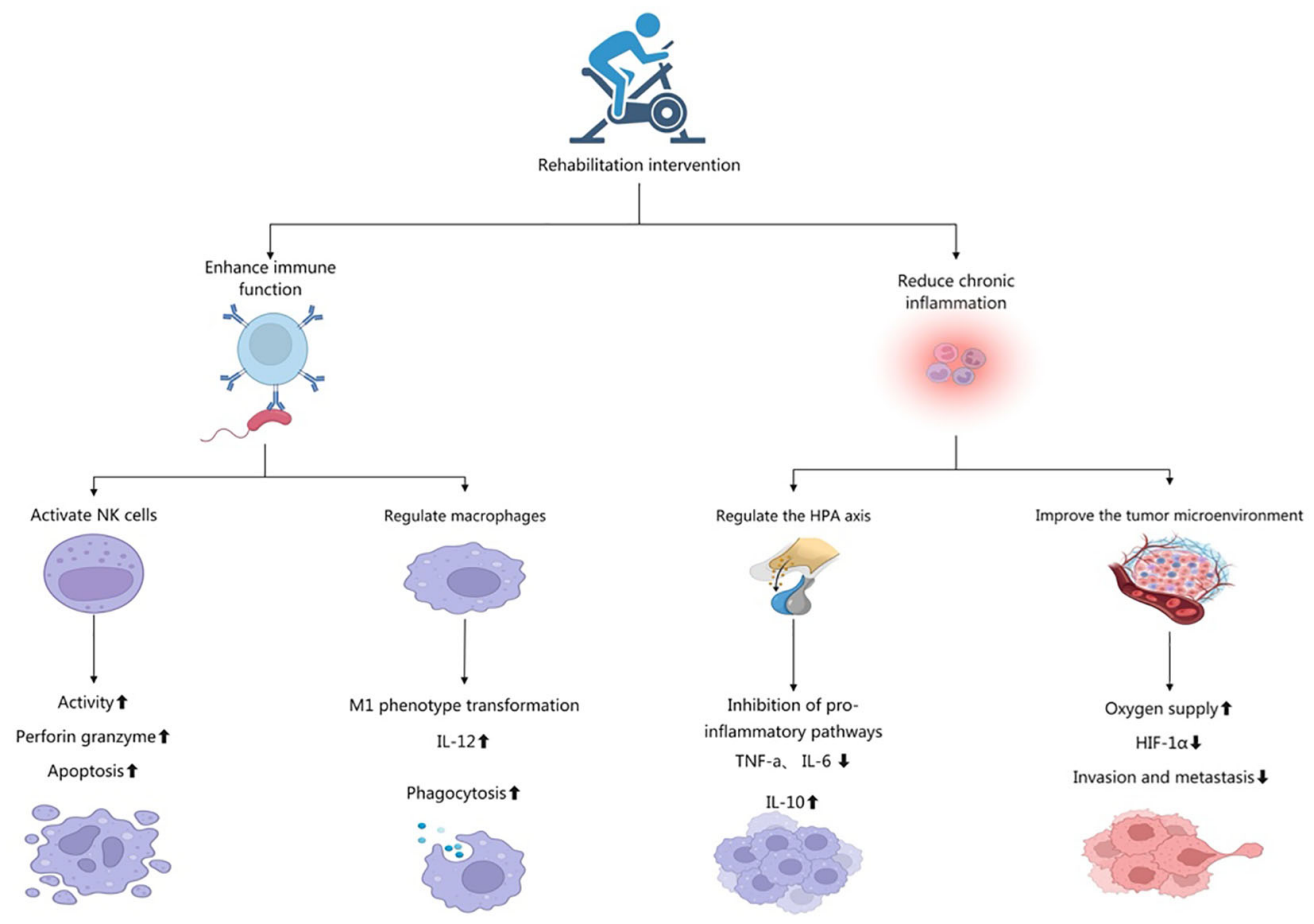
Mechanistic pathways of rehabilitation interventions in mitigating cancer metastasis. Rehabilitation modalities primarily exert anti-metastatic effects through dual mechanisms: immune modulation and the attenuation of chronic inflammation. On the immune axis, interventions activate NK cells to upregulate perforin and granzyme expression, thereby promoting tumor cell apoptosis. Simultaneously, they induce macrophage polarization toward the anti-tumor M1 phenotype, enhancing IL-12 secretion and phagocytic capacity. Concurrently, on the inflammatory axis, rehabilitation regulates the HPA axis to inhibit pro-inflammatory cytokines while upregulating anti-inflammatory IL-10. This collectively improves the TME by increasing local oxygen supply and downregulating HIF-1α ultimately suppressing tumor invasion and metastasis. Created by the authors using EdrawMax.

Moreover, frequent exercise significantly enhances the hypoxic condition of the TME (18). Hypoxic circumstances often facilitate the invasion and spread of tumor cells, whereas exercise enhances tissue oxygenation and improves local microcirculation, thus decreasing the expression of hypoxia-inducible factor (HIF-1α) (38)and decelerating tumor progression (**Figure 2**).

### 3.2 Determinants influencing tumor biomarker expression

Targeted rehabilitation techniques, including exercise interventions, exert direct physiological effects by modulating hormone levels and cytokine expression, thereby directly influencing tumor growth and metastatic potential. Meanwhile, psychosocial support indirectly benefits patients by improving psychological well-being, which can further modulate stress pathways and support immune function (39). Specifically, these interventions may reduce circulating pro-tumorigenic factors such as insulin-like growth factor-1 (IGF-1) and inflammatory cytokines, while increasing anti-inflammatory mediators, thus creating a less favorable environment for tumor cell proliferation and dissemination. These therapies enhance patients’ physiological condition-such as improving immune surveillance and preserving or increasing skeletal muscle mass and function-and are essential in modulating the TME. Exercise intervention impacts the TME by modulating the endocrine system (18). Consistent rehabilitative exercises, like brisk walking, swimming, and yoga, can enhance the secretion of endorphins in the body (40). Endorphins, functioning as an endogenous analgesic and anti-stress agent, mitigate patients’ stress, alleviate pain, and enhance their general psychological well-being (41).

Moreover, elevated endorphin levels have been associated with improved immune function and reduced systemic inflammation, both of which are critical in supporting cancer therapy and rehabilitation (22). By mitigating cancer-related fatigue and enhancing mood, these neuroendocrine improvements may also contribute to better adherence to rigorous treatment protocols and potentially lower the risk of cancer recurrence. Furthermore, consistent exercise positively regulates metabolic adaptations and normalizes blood circulation within the TME, alleviating local hypoxia and thereby suppressing metastatic progression (18). Exercise enhances local blood circulation and tissue oxygenation, hence decreasing hypoxia levels in tumor tissues (42). Hypoxia is a significant characteristic of the TME, and can enhance the proliferation and metastatic potential of tumor cells by upregulating HIF-1α. HIF-α is a key transcription factor activated under low oxygen conditions, which promotes angiogenesis, metabolic adaptation, and the expression of genes that support tumor cell survival, proliferation, and metastasis. Exercise intervention can diminish HIF-1α expression by enhancing local tissue oxygenation, thereby reducing tumor cell invasiveness (43). Research indicates that suitable exercise can modulate the expression of adhesion molecules on tumor cell surfaces, diminish the attachment capacity of tumor cells to the surrounding matrix, and inhibit their distant dissemination via the arterial or lymphatic systems (44).

**Figure 3:**
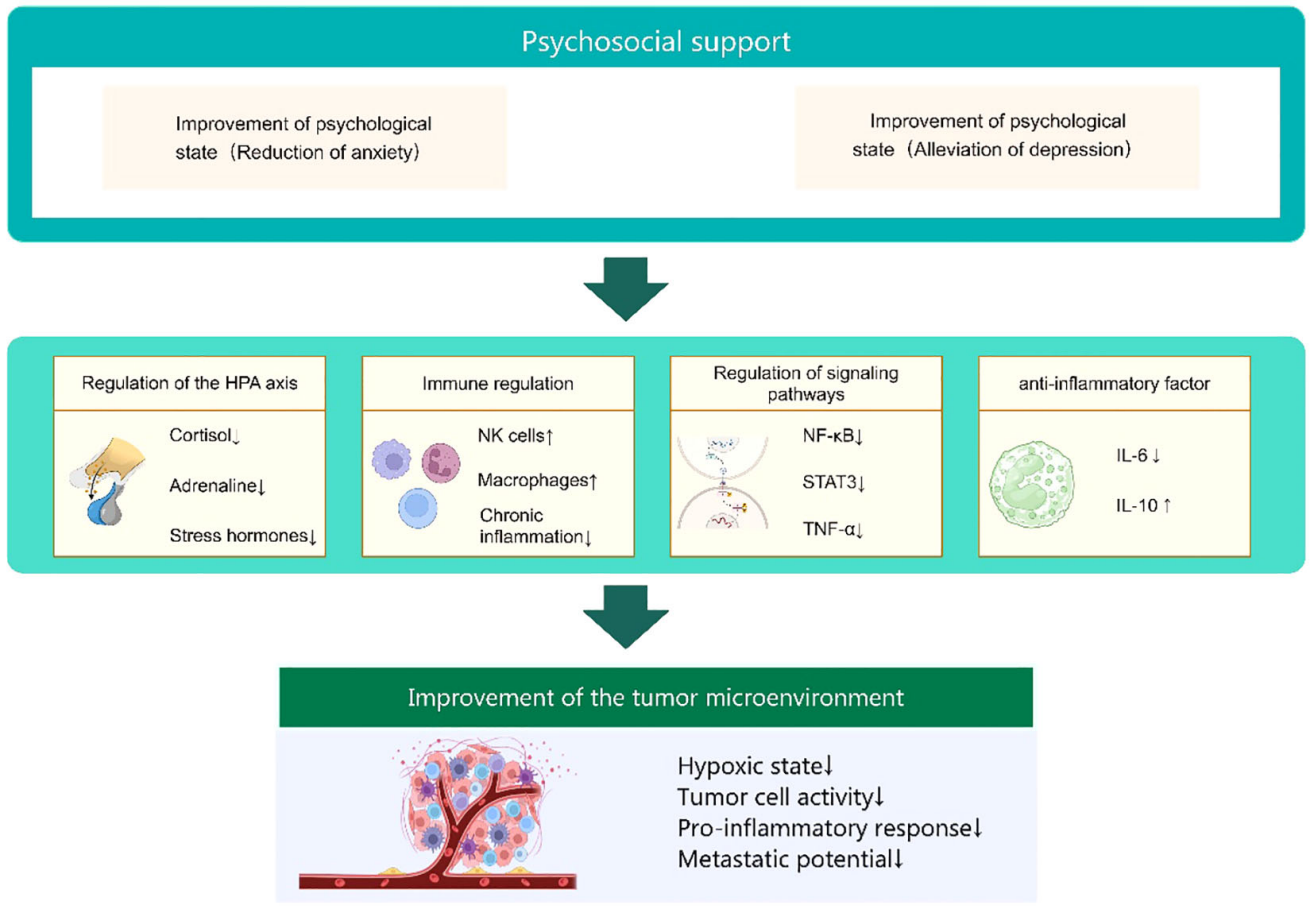
Psychophysiological pathways linking psychosocial interventions to the inhibition of cancer metastasis. Psychosocial support mitigates psychological distress translating these psychological improvements into systemic anti-tumor physiological responses. Central to this mechanism is the attenuation of the HPA axis, leading to reduced circulating levels of stress hormones such as cortisol and adrenaline. This neuroendocrine modulation directly suppresses pro-oncogenic intracellular signaling pathways, specifically NF-κB and STAT3, resulting in the downregulation of pro-inflammatory cytokines and the upregulation of anti-inflammatory mediators. Collectively, these systemic shifts enhance immune surveillance by boosting NK cell and macrophage activity. Consequently, this multi-target regulation ameliorates the TME by alleviating hypoxia and dampening local inflammatory responses, ultimately diminishing tumor cell viability and metastatic potential. Created by the authors using EdrawMax.

Psychological intervention indirectly influences the TME by modulating patients’ psychological states (45). Chronic psychological stress can stimulate the HPA axis, resulting in the overproduction of stress hormones and the enhancement of pro-inflammatory responses (46). Specifically, chronic stress increases the secretion of cortisol and elevates levels of pro-inflammatory cytokines such as IL-6 and TNF-α. This creates a tumor-promoting microenvironment by suppressing anti-tumor immunity and facilitating tumor growth and progression (47, 48). Effective psychosocial assistance, including cognitive behavioral therapy, mindfulness therapy, and emotional support groups, can ease patients’ anxiety and despair, thereby reducing cortisol and adrenaline levels and diminishing the expression of stress-related biomarkers (49). This enhancement in psychological condition aids in diminishing the activation of pro-inflammatory signaling pathways, thereby regulating the endocrine system and suppressing the activity and spreading potential of tumor cells (50). Moreover, psychosocial support can augment patients’ positive psychological coping, enhance their general health status, and further facilitate the proper functioning of the immune system and the amelioration of the TME (51). These extensive rehabilitation interventions markedly diminish the likelihood of tumor proliferation and metastasis by modifying multiple facets of the TME, therefore enhancing patients’ long-term survival rates and quality of life (52) (**Figure 3**).

### 3.3 Regulation of hormonal concentrations in the body

Rehabilitation intervention modulates hormonal levels in the body via many pathways and is crucial in inhibiting tumor spread (53). The two primary components of rehabilitation intervention are psychological and social support, together with frequent rehabilitation exercises (54). By modulating stress hormones, sex hormones, and facilitating the production of beneficial neurotransmitters, it markedly enhances the physical and mental condition of patients, reinstates immunological function, and diminishes the likelihood of tumor spreading (55). The control of stress hormones constitutes a fundamental mechanism in rehabilitation interventions (18). The stress response is typically governed by the HPA axis, with cortisol being one of its ultimate products (56). Cortisol is a crucial hormone associated with stress (57). Prolonged excessive secretion can induce a range of detrimental effects on the immune system, including the suppression of immune cell proliferation and activity, as well as the enhancement of pro-inflammatory cytokine production, such as TNF-α and IL-6, which exacerbates the TME and facilitates the growth and metastasis of tumor cells (58).

Psychological and social interventions, including counseling, group therapy, and the creation of support networks, can substantially alleviate patients’ psychological stress (59). Psychological stress is the primary catalyst for increased cortisol release (60). These intervention approaches significantly ease patients’ anxiety and despair, restore balance to the hyperactive HPA axis, and normalize cortisol levels. Research indicates that prolonged elevated cortisol levels result in immunological suppression and intensify inflammatory responses by activating the NF-κB signaling pathway, which is a key regulator of inflammation, so facilitating the survival and spread of tumor cells (61). Psychological intervention diminishes cortisol output and obstructs pro-inflammatory signaling pathways, thereby mitigating the influence of chronic inflammation on tumor spread (62). On the other hand, aerobic exercise and weight training stimulate the secretion of beneficial hormones in the body. Aerobic exercise and weight training stimulate the secretion of beneficial hormones in the body, including endorphins and serotonin (63). These beneficial hormones are essential for enhancing patients’ mental well-being. Endorphins, being natural painkillers produced within the body, provide analgesic and euphoric properties, successfully mitigating pain, enhancing mood, and decreasing discomfort associated with tumor therapy (64). The release of endorphins markedly elevates after consistent aerobic activities (such as jogging and swimming) and resistance training, aiding patients in managing psychological and physiological stress during therapy (65). Serotonin is a neurotransmitter integral to emotional regulation, significantly influencing mood and mitigating anxiety and sadness (66). Moderate exercise elevates the synthesis and release of serotonin, hence increasing patients’ mood and indirectly strengthening immune surveillance performance. The elevation of serotonin diminishes the activity of the HPA axis and lowers cortisol release, thereby alleviating the inhibitory impact on the immune system (67).

Furthermore, rehabilitative exercise is crucial for sustaining the equilibrium of sex hormones, particularly in regulating testosterone and estrogen levels (68). These hormones not only play crucial roles in reproductive activities but also exert major influence on immune system control (69). An excessive imbalance of testosterone and estrogen, particularly during tumor therapy, might adversely affect the immune system by suppressing T cell and B cell activities, thereby diminishing immunological surveillance (70, 71). Research indicates that modest rehabilitative exercise can sustain the equilibrium of sex hormone levels in the body (72). For instance, weight training can elevate testosterone release, which not only improves muscle strength and physical fitness but also augments NK cell activity through its immunological regulatory impact, facilitating their detection and destruction of tumor cells (73). Moreover, consistent aerobic activity might positively influence estrogen metabolism by decreasing body fat percentage and mitigating excessive estrogen accumulation in the body (74). Elevated estrogen levels may facilitate the development of specific malignancies, including breast cancer (75). Consequently, rehabilitation exercises may indirectly impede the advancement and metastasis of malignant malignancies by regulating estrogen levels.

**Figure 4:**
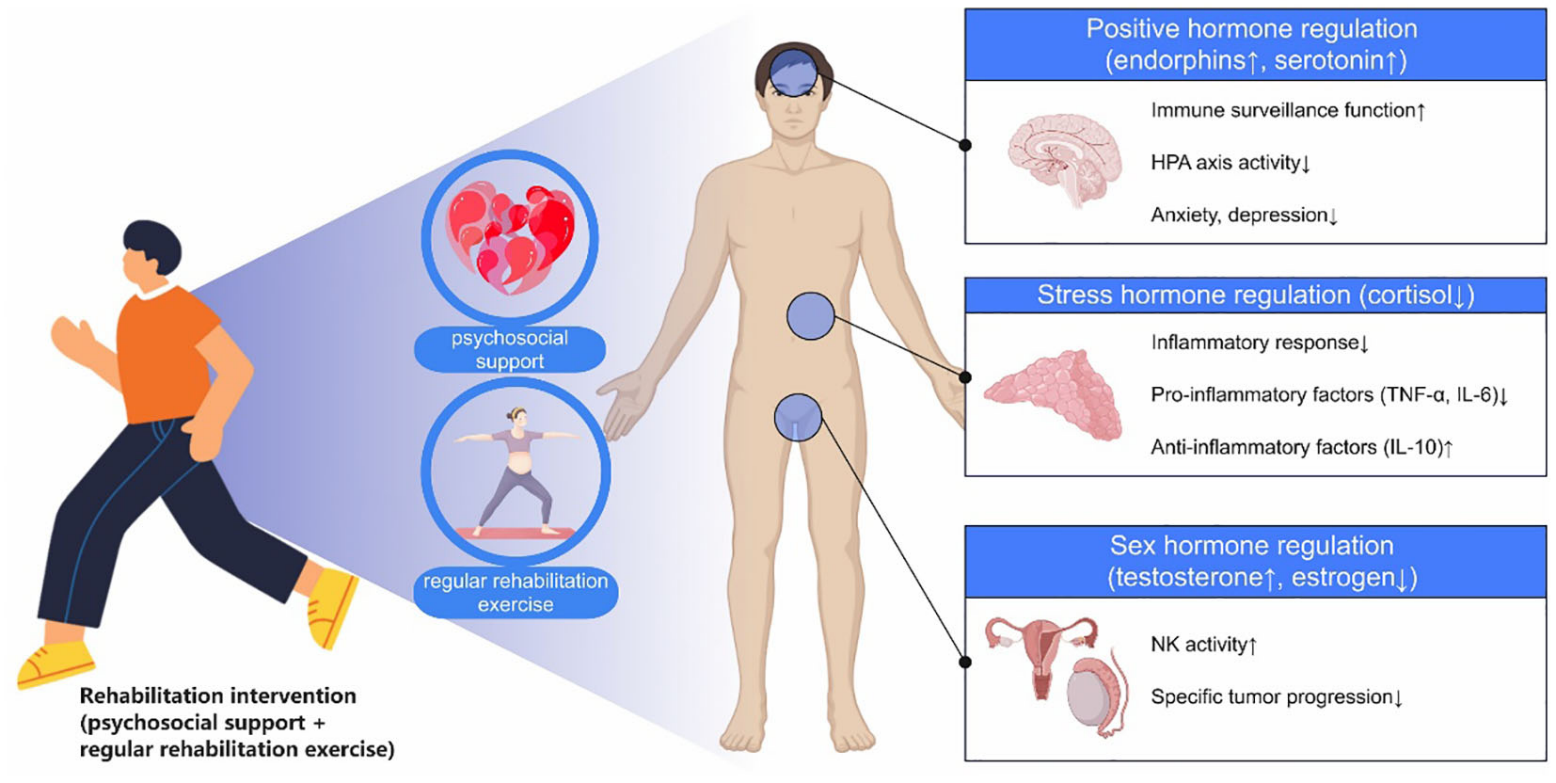
Systemic neuroendocrine and immune modulation driven by comprehensive rehabilitation interventions. The integration of psychosocial support and regular rehabilitation exercise orchestrates a systemic anti-tumor environment via the neuroendocrine-immune network. In the central nervous system, these interventions upregulate positive neuromodulators, which alleviates anxiety and depression while dampening the hyperactive HPA axis. This neuroendocrine shift reduces the secretion of stress hormones, particularly cortisol, thereby mitigating systemic inflammatory responses evidenced by the downregulation of pro-inflammatory cytokines and the upregulation of anti-inflammatory IL-10. Furthermore, the regulation of sex hormones synergizes with these processes to augment NK cell cytotoxicity. Collectively, this restoration of endocrine homeostasis and immune surveillance robustly inhibits tumor progression and metastatic dissemination. Created by the authors using EdrawMax.

Rehabilitation intervention offers patients an improved endocrine milieu by modulating several hormones, including cortisol, endorphins, serotonin, and sex hormones, therefore reinstating and augmenting immune system functionality. The management of hormone levels efficiently inhibits tumor spread by enhancing the TME, suppressing pro-inflammatory pathways, and facilitating the generation of anti-inflammatory cytokines (**Figure 4**).

## 4 Rehabilitation intervention strategies

### 4.1 Types of rehabilitation interventions

Rehabilitation interventions for cancer patients encompass diverse strategies aimed at improving physical function and overall health, while also affecting the TME, immune system, and anti-inflammatory mechanisms through multidimensional regulatory effects. Aerobic exercise and resistance training, as crucial elements of rehabilitation in exercise therapy, exhibit considerable anti-cancer efficacy (76). Aerobic exercise can efficiently modulate the immune system, specifically by augmenting the activity of T cells and NK cells, thus strengthening the body’s capacity to identify and eradicate tumor cells (77). Aerobic exercise enhances blood circulation and oxygen delivery, alleviating the effects of hypoxia in the TME and thereby diminishing the likelihood of tumor cell spread (78). Aerobic exercise can inhibit tumor-associated chronic inflammation and diminish the synthesis of pro-inflammatory cytokines (such as TNF-α and IL-6), hence lowering the chance of cancer spread (79). Resistance exercise can impede the proliferation of cancer cells by enhancing muscular mass, strength, and metabolic function (80, 81). Resistance training can modulate metabolic pathways, improve mitochondrial function, and diminish the invasive capacity of tumor cells via aberrant energy metabolism (82). Exercise therapy enhances patients’ general physical fitness while significantly diminishing the chance of tumor dissemination and metastasis through the regulation of immune responses, anti-inflammatory mechanisms, and metabolic processes (83, 84). Customized exercise regimens in physical therapy can augment muscular strength, enhance joint functionality, and sustain functional mobility by mitigating pain and activity restrictions resulting from cancer metastasis, thereby contributing to the prevention of tumor dissemination (85). Rehabilitation interventions aimed at the lymphatic system, such as manual lymphatic drainage, are especially beneficial for breast cancer patients, markedly decreasing the incidence of lymphedema and diminishing the likelihood of cancer cell proliferation via the lymphatic system by facilitating lymphatic fluid return (86) (**Table 1**).

**Table 1:** Structured exercise interventions in cancer rehabilitation.

Modality	Frequency	Intensity	Time	Type	Outcomes	Mechanisms
Aerobic (14)	3–5 times/week	Moderate (40–60% VO_2_max or 3–6 METs)	20–45 min/session	Walking, cycling, treadmill	Cardiorespiratory fitness, fatigue, QoL, immune markers	↓Inflammation, ↑NK cell function
Resistance (87)	2–3 times/week	Moderate (60–70% 1RM)	20–45 min/session	Free weights, resistance bands	Strength, muscle mass, function	↑Muscle protein synthesis, ↑Insulin sensitivity
Combined (88)	2–3 times/week	Moderate	40–60 min/session	Aerobic +resistance	Combined above outcomes	Synergistic effect: function, body composition
Flexibility/Stretching (89)	2–3 times/week	To point of tightness	10–20 min/session	Static or dynamic stretches	Range of motion, pain, stiffness	Maintains joint mobility, ↓musculoskeletal discomfort
Mind–body (Yoga,Tai Chi) (90)	1–3 times/week	Low–moderate	30–60 min/session	Yoga, Tai Chi, Qigong	Anxiety, sleep, QoL, fatigue	↓Cortisol, ↑Vagal tone

Recent research has progressively demonstrated the synergistic effects of exercise treatment and physical factor intervention in suppressing tumor metastasis. During the acute postoperative phase, preclinical research indicates that high-intensity interval training (HIIT) can augment the tumor-eradicating capacity of CD8+ T cells by stimulating the secretion of myokines, such as IL-15, from muscle tissue (91). Myokines are cytokines or peptides released by muscle fibers during contraction, which can modulate immune cell activity. For example, IL-15 enhances the proliferation and cytotoxicity of CD8+ T cells and NK cells, thereby strengthening the body’s anti-tumor immune response (92). Concurrently, local hyperthermia has been demonstrated to elevate the expression of HSP70 on the surfaces of tumor cells, thereby improving the recognition efficacy of NK cells for circulating tumor cells (93). Clinical cohort studies suggest that the combination of the two can decrease the incidence of cancer metastases following cancer surgery (94). In patients with stable disease, moderate-intensity continuous training has been shown to diminish tumor-associated macrophage infiltration by inhibiting the HMGB1/TLR4 signaling pathway in breast cancer models (95), whereas transcutaneous electrical nerve stimulation (TENS) preserves NK cell activity by modulating the β-endorphin/μ-opioid receptor axis and alleviating pain-related stress responses (96). The amalgamation of the two markedly enhances the 1-year progression-free survival rate of patients with non-small cell lung carcinoma (97). In the long-term rehabilitation phase, the efficacy of resistance training in postponing cancer cachexia via the IGF-1/mTORC1 pathway has been corroborated by numerous randomized controlled trials (98). The insulin-like growth factor 1 (IGF-1)/mechanistic target of rapamycin complex 1 (mTORC1) pathway constitutes a central cellular signaling cascade governing skeletal muscle homeostasis. Activation of this axis robustly upregulates muscle protein synthesis and concurrently suppresses proteolytic pathways, thereby effectively inhibiting muscle wasting and mitigating cancer-related cachexia (18, 99). While the identification of whole-body vibration therapy as a means to impede YAP/TAZ nuclear translocation through the activation of the mechanically sensitive ion channel PIEZO1 presents a novel target for obstructing mechanically induced metastatic spread (100). YAP (Yes-associated protein) and TAZ (transcriptional coactivator with PDZ-binding motif) are critical mechanosensitive transcriptional coactivators. In a pro-tumorigenic microenvironment, mechanical cues trigger YAP/TAZ to translocate into the cell nucleus, where they bind to transcription factors to drive the expression of target genes responsible for tumor cell proliferation, EMT, and metastasis. Impeding this nuclear translocation effectively sequesters YAP and TAZ in the cytoplasm, neutralizing their pro-metastatic signaling cascades (101). PIEZO1 is a mechanically sensitive ion channel found in cell membranes that responds to mechanical stimuli, and its activation can influence cell signaling and movement, thereby affecting the metastatic potential of tumor cells. Current evidence indicates that exercise intervention mostly influences systemic immune regulation, whereas physical factor therapy emphasizes the remodeling of the local microenvironment (18). The sequential integration of the two may yield a synergistic impact of “1 + 1>2”; nevertheless, the molecular docking mechanism and personalized dosing necessitate further investigation through interdisciplinary research (102).

Alongside exercise therapy and physical factor treatment, various rehabilitation treatments, as crucial elements of rehabilitation intervention, significantly contribute to the overall enhancement of patients’ quality of life and health status. Occupational therapy assists patients in adjusting to physical limitations and enhancing their capacity to execute daily activities, such as dressing, eating, and personal hygiene management (103). This, in turn, mitigates life stress and modulates the immune system by alleviating psychological burdens, which may inhibit cancer metastasis (104). Pain management, a crucial component of rehabilitation, particularly in physical therapies such as electrotherapy, cryotherapy, thermotherapy, and massage, enhances patient mobility by modulating the nervous system and alleviating chronic pain, diminishing the body’s stress response, and consequently inhibiting the proliferation and metastasis of tumor cells via stress-related mechanisms (105). Furthermore, the use of extensive rehabilitation strategies from traditional Chinese medicine, including acupuncture and herbal interventions, enhances the modalities of rehabilitation treatment (106). Acupuncture modulates the central nervous system and immunological response, alleviates pain, and enhances sleep quality (107). It may also impede metastatic factors in the TME by modulating the production of HPA axis and inflammatory mediators (108). Herbs such as Astragalus and Ganoderma lucidum exhibit substantial immune regulatory properties, augmenting macrophage and T cell capabilities, strengthening the body’s immune surveillance capacity, and diminishing the proliferation and metastasis of cancer cells (109). Long-term stress and negative emotions are significantly associated with cancer spread in the context of psychological and social support (110). Psychological interventions, including cognitive behavioral therapy (CBT) and mindfulness-based stress reduction (MBSR), can modulate patients’ psychological states and endocrine systems, mitigate the adverse effects of chronic stress on the TME, and effectively impede the proliferation and migration of tumor cells (111, 112). Consequently, rehabilitation interventions, utilizing multi-dimensional processes, exhibit extensive and significant effects in preventing tumor metastasis, thereby offering enhanced support and safeguarding the long-term health of cancer patients (**Table 2**).

**Table 2:** Types of rehabilitation interventions and their mechanisms of action.

Intervention type	Specific forms of intervention	Main mechanism of action
Exercise therapy	Aerobic exercises (jogging, swimming, brisk walking)	Regulate immune function; increase blood circulation and oxygen supply; reduce the risk of metastasis (84, 113).
	Resistance training (strength training, resistance exercise)	Enhance metabolic function; delay cancer cachexia; inhibit tumor invasion (114).
	HIIT	Improve blood circulation and promote the remodeling of the TME (115).
Physical factor therapy	Local hyperthermia	Upregulate the expression of HSP70 on the surface of tumor cells to enhance the recognition efficiency of circulating tumor cells (116).
	TENS	Reduce pain-related stress responses; protect NK cell activity and reduce chronic inflammation activation (117).
	Whole body vibration therapy	Inhibit mechanical transfer and block the mechanical force-driven metastasis and dissemination (118).
Occupational therapy	Daily activity training	Reduce psychological burden; enhance functional activity ability; relieve pain and activity limitation caused by tumor metastasis (103).
Pain management	Electrotherapy, cold and heat therapy	Alleviate pain and stress responses, reduce the promoting effect of chronic pain on tumor metastasis; decrease the activation of pro-inflammatory pathways (119).
Traditional Chinese Medicine rehabilitation	Acupuncture	Regulate the HPA axis, reduce the secretion of stress hormones; inhibit pro-metastatic factors: control the secretion of inflammatory mediators, suppress pro-metastatic factors in the TME (107).
	Chinese herbal medicines (such as Astragalus membranaceus and Ganoderma lucidum, etc.)	Enhance immune function, improve the body’s immune surveillance ability, and reduce the risk of cancer cell spread (109).
Psychotherapy	CBT, MBSR	Relieve anxiety and depression; reduce HPA axis activation, decrease cortisol secretion, and inhibit chronic inflammation; suppress the deterioration of the TME: reduce the promotion of tumor cell migration and proliferation by negative emotions (111, 112).

### 4.2 Rehabilitation intervention strategies for various transfer locations

The metastasis of cancer is a critical phase in the advancement of the disease. Various cancer forms exhibit distinct proclivities for metastasis, targeting sites such as the bone, lung, liver, brain, lymph nodes, skin, and gastrointestinal system (120). For these metastatic sites, comprehensive rehabilitation therapies aim not only at functional recovery and pain management but also at preventing and mitigating tumor dissemination by modulating the TME and employing anti-inflammatory and immunological modulation mechanisms.

Rehabilitation options for bone metastases encompass physical therapy, pain management, functional training, and nutritional support (121). These treatments seek to fortify the musculature around the bones, strengthen the skeletal framework, and alleviate pain resulting from bone degradation (122, 123). Simultaneously, exercise and nutritional intervention can modulate the levels of inflammatory mediators like IL-6 and TNF-α, diminish the pro-inflammatory response within the bone microenvironment, and minimize the retention and invasion of tumor cells in bone tissue (124, 125). Moreover, suitable dietary assistance, including sufficient calcium and vitamin D, can facilitate the restoration of bone tissue by preserving bone density and improving immunological function, while mitigating the detrimental effects of tumor cells on the bone (126). Rehabilitation interventions for lung metastases concentrate on the modulation of respiratory function and the immune system. Respiratory training and aerobic exercise promote lung function and cardiovascular endurance, prevent lung infections, and augment the activity of T cells and NK cells, hence increasing immune surveillance in lung tissue and limiting cancer cell metastasis (127). Aerobic exercise enhances the body’s oxygen usage efficiency, ameliorates the hypoxic conditions in the lungs, and diminishes the proliferation rate of tumor cells in hypoxic environments (128). Additionally, chest physical therapy enhances respiratory muscle power, alleviates airway blockage, consequently augmenting lung airflow, diminishing local inflammation, and decreasing the likelihood of tumor cell dissemination via the vascular and lymphatic systems (129).

The rehabilitation options for liver metastases mostly emphasize immunological function and metabolic homeostasis (130). High-protein, low-fat dietary support aids in preserving the patient’s weight and nutritional status, bolsters the immunological response, particularly by augmenting the activity of anti-tumor immune cells (such as macrophages and T cells), and improves the capacity to eliminate tumor cells (131). Simultaneously, moderate physical training enhances hepatic blood circulation, fosters hepatocyte regeneration, modulates oxidative stress within the hepatic milieu, diminishes hepatocellular damage, and impedes tumor cell proliferation (132). Furthermore, consistent monitoring of liver function can effectively avert worsening and assist in optimizing the rehabilitation intervention strategy (133).

The rehabilitation strategy for brain metastases concentrates on restoring neurological function, enhancing cognitive ability, and managing pain and epilepsy (134). Physical therapy and occupational therapy facilitate the restoration of motor coordination, enhance cerebral blood flow, promote neural plasticity, and diminish the accumulation of inflammatory mediators such as IL-1β and TNF-α in the brain, thereby mitigating neurological damage resulting from tumor metastasis (135, 136). Cognitive training can assist patients in preserving or recovering brain function, enhancing cognitive abilities, and modulating the brain’s neural network to mitigate tumor-associated neurological impairments (137). Pain management and epilepsy prevention further mitigate pro-metastatic elements in the TME by diminishing neuroinflammation and excessive excitatory responses, while controlling neurotransmitter balance (138). Rehabilitation options for lymph node metastases encompass early detection and surveillance, complete therapy, and physical intervention (139). Advanced lymphatic drainage procedures actively facilitate the clearance of interstitial fluid and effectively mitigate lymphedema. Crucially, contemporary oncological consensus demonstrates that restoring lymphatic transport capacity does not exacerbate the risk of tumor cell dissemination. Instead, normalizing local lymphatic flow is postulated to alleviate interstitial fluid pressure-induced immunosuppression, thereby helping to restore immune surveillance within the regional lymph nodes and maintaining overall oncological safety (140). Moreover, suitable exercise interventions can stimulate immune cells inside the lymphatic system, augment the body’s immune surveillance, and inhibit the proliferation and dissemination of tumor cells (141). Targeted lymphatic rehabilitation therapies, such as manual lymphatic drainage and decongestive interventions, play a fundamental role in modulating local immune responses and resolving chronic tissue inflammation (142). By actively facilitating the clearance of interstitial fluid stasis, these therapies diminish the local accumulation of pro-inflammatory cytokines and alleviate mechanical stress within the tissue (143). Consequently, the restoration of physiological lymphatic function helps disrupt the chronic inflammatory pathways within the TME that would otherwise foster immune evasion and promote tumor dissemination, thereby reinforcing regional anti-tumor immune surveillance (144).

Rehabilitation for metastasis in the skin and gastrointestinal tract integrates local treatment, nutritional support, and physical training to modulate the immune response and inflammatory condition, thereby aiding in tumor growth control (145). Localized interventions, including dermatological care and physical modality therapy, can diminish local inflammatory responses and restore skin barrier integrity, therefore mitigating the danger of tumor dissemination in the dermis and gastrointestinal system (146, 147). Nutritional assistance facilitates nutrition absorption in the gastrointestinal tract and sustains digestive system function, hence improving the patient’s physical state and aiding in the equilibrium of the TME (148). Simultaneously, physical exercise improves the body’s antioxidant capacity, diminishes inflammatory responses, and strengthens the patient’s general immunity and cancer resistance (14).

Rehabilitation interventions for various metastatic cancer sites, utilizing interdisciplinary collaboration and tailored treatment programs, effectively modulate the TME, immunological function, and inflammatory response (149). They not only attain favorable outcomes in the restoration of physical functions but also proficiently impede the onset and advancement of tumor metastasis through multifaceted pathways, including immunological modulation, anti-inflammatory processes, and metabolic management. Through ongoing assessment of rehabilitation outcomes, alongside patient education and self-management, rehabilitation interventions can offer more tailored and holistic support for cancer patients, enhancing their long-term survival rates and quality of life while facilitating the overall recovery of their physical and mental well-being (**Tables 3**, **4**).

**Table 3:** Rehabilitation intervention strategies for various transfer location.

Metastatic site	Intervention measures	The main mechanism of action
Bone metastasis	Physical therapy: functional training, strength training Nutritional support	Stabilize bone structure and enhance muscle strength; reduce pro-inflammatory factors (IL-6, TNF-α) Maintain bone density (118, 121, 131).
Pulmonary metastasis	Breathing exercises Aerobic exercise	Improve lung function and enhance immune surveillance; relieve hypoxic conditions and inhibit tumor spread (51, 97, 103, 114).
Liver metastasis	High-protein, low-fat diet Low to moderate intensity exercise	Enhance immune function and improve the ability to resist tumor cells; regulate the liver microenvironment and reduce oxidative stress (130, 133).
Brain metastasis	Physical therapy Cognitive training Pain management	Improve neural function and promote neural regeneration; reduce neural inflammation and regulate the balance of neurotransmitters (134, 135, 137).
Lymph node metastasis	Lymphatic drainage Moderate exercise	Promote lymphatic circulation; activate immune cells and reduce the risk of spread (139).
Skin metastasis	Local care Nutritional support	Repair the skin barrier and reduce local inflammation, enhance the body’s immune capacity (145, 148).
Gastrointestinal metastasis	Nutritional support Light physical training	Maintain digestive function and enhance physical fitness; reduce gastrointestinal inflammation and inhibit tumor spread (108, 130).

**Table 4:** Comparative summary of clinical trials of rehabilitation interventions on cancer metastasis.

Intervention	Cancer type	Study design	Outcome on metastasis	Level of evidence
Aerobic exercise (moderate)	Breast, Prostate, Colorectal	RCT/Meta-analysis (150, 151)	No significant reduction in metastasis incidence; improvement in immune markers and QoL	Level 1 (high)
Resistance training	Breast, Hematological	RCT/Systematic Review (152)	No direct effect on metastasis; improves muscle mass, physical function	Level 1 (high)
HIIT	Breast, Animal models	Preclinical/Clinical Cohort (11)	In animal models: reduced lung metastasis and enhanced CD8+ T cell activity; human evidence limited	Level 2 (moderate)
Combined Aerobic + Resistance	Mixed solid tumors	RCT (153)	Improved functional status; no significant effect on metastasis-free survival	Level 1 (high)
Physical factor (Hyperthermia)	Breast,GI (mostly preclinical)	Preclinical/Clinical (154)	Enhanced immune surveillance, reduced local recurrence, limited direct evidence on metastasis	Level 2 (moderate)
TENS (pain management)	Lung, Mixed	Pilot Clinical/Animal (155)	Improved NK cell function, reduced stress; insufficient direct evidence on metastasis	Level 2 (moderate)
Mind-body (Yoga, Tai Chi, CBT)	Breast, Colorectal, Lung	RCT/Systematic Review (156)	Reduced inflammation and stress hormones, no direct evidence for metastasis	Level 1 (high)

RCT, Randomized Controlled Trial; QoL, Quality of Life; HIIT, High-Intensity Interval Training; GI, Gastrointestinal; TENS, Transcutaneous Electrical Nerve Stimulation; NK, Natural Killer; CBT, Cognitive Behavioral Therapy.

### 4.3 Cancer type and patient-specific considerations in rehabilitation interventions

Recent evidence underscores the importance of tailoring rehabilitation interventions according to cancer subtype, disease stage, and patient population. For hematological malignancies (such as leukemia, lymphoma, and myeloma), rehabilitation protocols emphasize infection prevention, fatigue management, and safe mobilization due to immunosuppression and cytopenias, as described in the ACSM (American College of Sports Medicine) and NCCN (National Comprehensive Cancer Network) guidelines (157, 158). In contrast, for solid tumors (e.g., breast, lung, colorectal), rehabilitation strategies are often disease- and stage-specific, focusing on surgical recovery, management of treatment-induced sequelae, and long-term functional maintenance (159). For instance, resistance and aerobic training are recommended for breast cancer survivors to address lymphedema and improve upper limb function, whereas for advanced lung cancer patients, pulmonary rehabilitation is prioritised (157).

Rehabilitation interventions should also be adapted to the phase of oncologic treatment: prehabilitation (prior to surgery/chemotherapy) aims to optimize baseline function and psychological resilience; during treatment, exercise is often reduced in intensity and carefully monitored to manage fatigue and immunosuppression; post-treatment, interventions focus on restoration of physical fitness, management of chronic sequelae, and prevention of recurrence. These phase-specific recommendations are supported by consensus statements from international bodies such as ACSM and ESMO (European Society for Medical Oncology) (159, 160).

Demographic and clinical characteristics, including age, sex, baseline fitness, and comorbidities, substantially influence rehabilitation planning (161). For older adults, exercise prescription must be individualized to accommodate frailty, fall risk, and polypharmacy (162). Pediatric and adolescent cancer survivors require age-appropriate, developmentally targeted programs (163, 164). Sex differences in rehabilitation outcomes (e.g., bone health, muscle mass retention) have also been reported, necessitating gender-sensitive approaches (165). Furthermore, patients with comorbidities such as cardiac or respiratory disease should undergo multidisciplinary assessment to ensure safety and efficacy (158).

Several clinical guidelines and position statements provide evidence-based recommendations for rehabilitation across cancer types and patient subgroups, including: (1) ACSM Guidelines for Exercise in Cancer Survivors (157); (2) NCCN Survivorship Guidelines (158); (3) ESMO Clinical Practice Guidelines (159).

### 4.4 Stakeholder-oriented practical implications and messaging

This review is intended to inform and empower a diverse range of stakeholders involved in the management and support of patients with cancer who are at risk of metastasis. For clinical professionals such as physicians, nurses, and palliative care teams, our findings underscore the importance of early and proactive integration of rehabilitation interventions within oncology care pathways to improve patient outcomes and quality of life. Rehabilitation and palliative care specialists can utilize the synthesized evidence to develop individualized, stage-appropriate rehabilitation protocols. In addition, dietitians and allied health professionals are encouraged to collaborate in multidisciplinary teams to address the physical, nutritional, and psychosocial factors pivotal to metastasis prevention. Researchers in areas including personalized rehabilitation, oncology, exercise science, and integrative medicine will find this review highlights key research gaps and provides direction for future investigations. Most importantly, for patients and their families, our review emphasizes the significance of evidence-based rehabilitation strategies, encourages open communication with healthcare providers, and urges caution against adopting non-validated interventions that may be encountered on social media or other non-scientific sources. By specifically tailoring our key messages, we aim to facilitate the translation of current evidence into clinical decision-making, collaborative multidisciplinary care, research innovation, and patient empowerment.

## 5 Potential rehabilitation intervention strategies from an interdisciplinary approach

### 5.1 Integration of physical therapy and occupational therapy

The amalgamation of physical therapy and occupational therapy in contemporary rehabilitation medicine exemplifies a robust interdisciplinary synergy. Multi-dimensional intervention measures can substantially improve patients’ functional recovery and quality of life (166). Physical therapists and occupational therapists collaborate to create tailored rehabilitation regimens that address patients’ individual requirements, ultimately enhancing rehabilitation outcomes on both physiological and psychological dimensions (167, 168). The primary objective of physical therapy is to rehabilitate patients’ physical functions via structured movement and strength training (169). Detailed functional examinations identify functional impairments, emphasizing muscle strength, flexibility, balance, postural control, and cardiopulmonary endurance. To attain the objective of rehabilitating and augmenting patients’ motor functions, individualized exercise regimens are formulated, generally encompassing aerobic exercise, resistance training, balance training, and stretching (152). Physical therapy can effectively mitigate functional deficits resulting from surgery, trauma, or chronic diseases using various methods (170). Occupational therapy emphasizes patients’ autonomy and adaptation in daily activities, seeking to assist individuals in restoring their functions and achieving independent living (168). Occupational therapists formulate individualized occupational training plans by conducting comprehensive evaluations of everyday activities, encompassing fundamental self-care tasks (such as dressing, hygiene, and nutrition) as well as intricate job, familial, and social engagements (166). In this process, occupational therapists implement specialized assistive devices (such as adaptive utensils and long-handled tools) and modify the environment’s layout to enhance patients’ self-care capabilities and efficiency in daily tasks, even when their physical functions remain impaired (171). This alleviates the caregiving burden on patients and their families while simultaneously bolstering patients’ self-esteem and autonomy, therefore enhancing their mental well-being (168).

The comprehensive combination of physical therapy and occupational therapy encompasses the restoration of bodily functions, the increase of daily activity capabilities, and the improvement of patients’ mental health and social adaptability. By collaborating closely with physical therapists, occupational therapists can more effectively integrate the motor skills acquired in physical therapy into particular daily living tasks. For instance, improvements in lower limb strength and balance achieved through physical therapy can be directly applied to daily activities such as transferring from bed to chair, walking on uneven surfaces, or climbing stairs. Occupational therapists then incorporate these skills into individualized training for tasks like safely navigating the bathroom, preparing meals in the kitchen, or using public transportation, thereby enabling patients to perform essential self-care and community activities more independently and confidently. Occupational therapists modify the home and work surroundings of patients who have been bedridden or have restricted mobility for an extended period, optimizing the outcomes of physical therapy to improve self-care capabilities and social engagement (172). By assessing and modifying patients’ work settings, occupational therapists can significantly diminish the effects of functional impairments on employment, thus enhancing job efficiency and occupational engagement. This facilitates patients’ social reintegration and offers essential assistance for their employment resumption and restoration of social functions (173).

**Figure 5:**
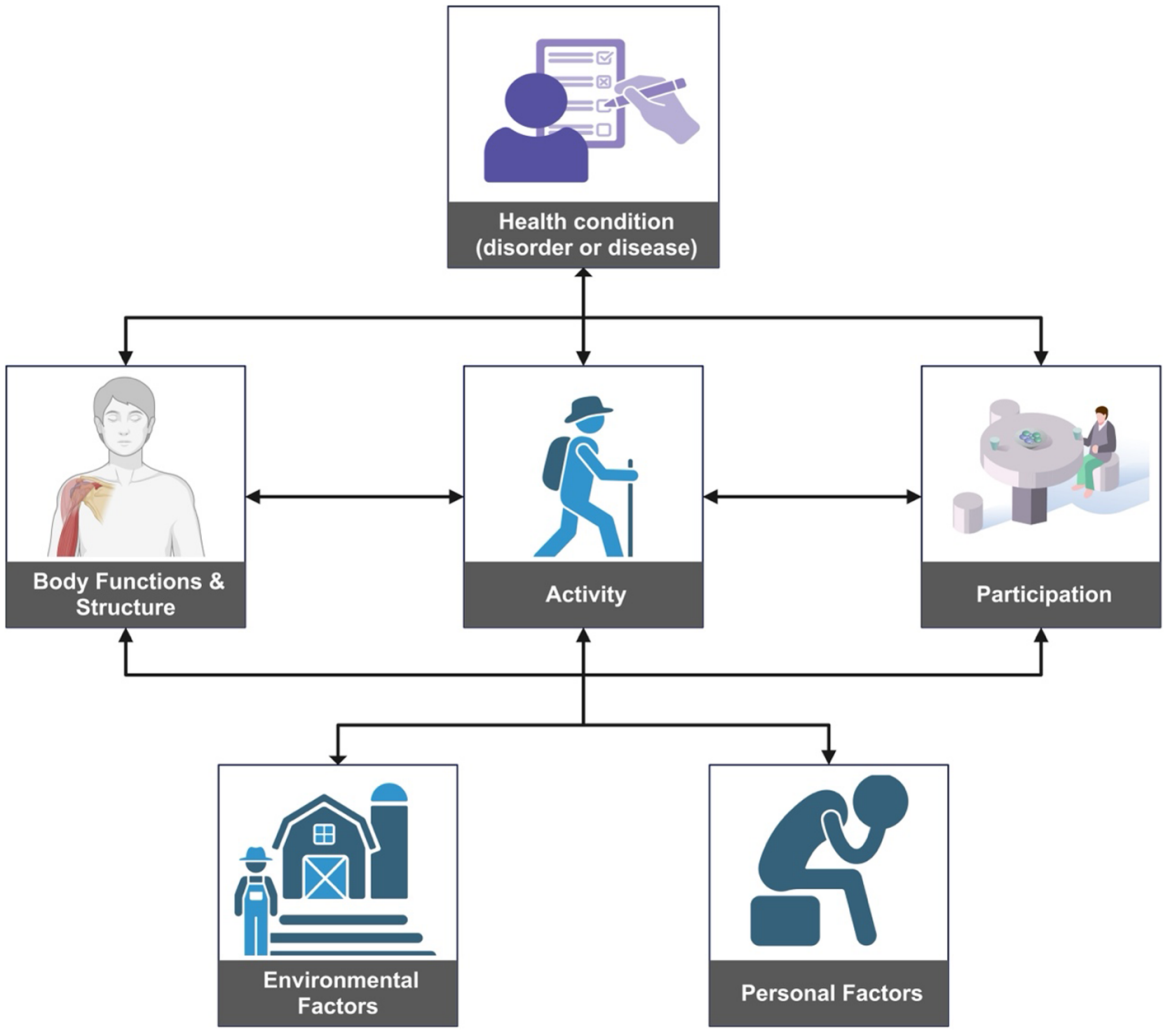
Conceptual framework of the biopsychosocial approach in cancer rehabilitation based on the WHO ICF model. This diagram depicts the dynamic and bidirectional interrelationships between a patient’s health condition and their overall functioning. The core domains of functioning include body functions and structure, activity, and participation. Crucially, these clinical and functional outcomes are continuously modulated by contextual elements, encompassing environmental factors and personal factors. In the context of cancer metastasis, this holistic framework guides multidisciplinary rehabilitation teams to design comprehensive, patient-centered interventions that not only mitigate biological impairments but also optimize functional independence and societal participation. Created by the authors using EdrawMax.

The amalgamation of physical therapy and occupational therapy in cancer rehabilitation must be predicated on a thorough evaluation within the context of the International Classification of Functioning, Disability and Health (ICF) (**Figure 5**). This framework offers a standardized instrument for assessing functional deficits in cancer patients across four dimensions: bodily functions and structures, activities and involvement, environmental factors, and personal aspects (174). Using postoperative upper limb lymphedema following breast cancer surgery as a case study, physical therapists employ the ICF framework to assess the extent of diminished shoulder joint range of motion and muscle strength deficits, subsequently formulating a graded compression treatment plan integrated with neuromuscular electrical stimulation based on environmental factors (175, 176). Concurrently, occupational therapy concentrates on evaluating activity limitations, such as fine motor tasks and tool utilization, utilizing a 3D motion capture system to detect compensatory postures, such as a cervical forward inclination angle exceeding 15°, during activities of daily living (ADL) like eating, grooming, and mobile phone usage, and then devises task-oriented training, such as adaptive utensil training, to mitigate the risk of joint strain (177). The ICF framework is pivotal for interdisciplinary collaboration: physical therapy addresses body function levels (e.g., enhancing postural transfer ability via whole-body vibration therapy) (178), whereas occupational therapy emphasizes the participation dimension (e.g., employing virtual reality to replicate a supermarket shopping environment to improve community activity participation) (179). Both disciplines align their intervention objectives by utilizing the ICF core data set. This indicates that using the bio-psycho-social model of the ICF into cancer rehabilitation can transcend the constraints of conventional medical metrics and effectively realize “function-oriented” precise intervention.

Physical therapy and occupational therapy are intricately interconnected and mutually reinforcing during all phases of the patient’s rehabilitation (166). In the acute rehabilitation phase, physical therapy aims to restore fundamental capabilities (178), whereas occupational therapy assists patients in preserving essential living skills through the utilization of assistive equipment (103). Upon entering the recovery phase, physical treatment augments the patient’s motor skills, while occupational therapy facilitates the gradual restoration of complex functions, including job and leisure activities, so facilitating societal reintegration (180). As rehabilitation advances, the interdisciplinary team consistently evaluates the patient’s functional development and adaptively modifies the rehabilitation plan according to their individual recovery status to optimize the attainment of rehabilitation objectives (181). This collaborative method enhances the patient’s comprehensive recovery while optimizing the efficiency of medical resource consumption through interdisciplinary teamwork (180).

### 5.2 The combination of nutrition and physical therapy

The integration of nutrition and physical therapy may play a significant role in accelerating the recovery process of patients, enhancing their overall health and quality of life (182). During the rehabilitation period, the body requires more energy and nutrients to support muscle repair, wound healing, and the restoration of the immune system (**Table 5**). Dietitians conduct detailed nutritional assessments to ensure that patients consume adequate and balanced nutrition to meet the physiological demands of recovery (183). Protein is a key element for muscle repair and growth, especially during physical therapy when muscles are stimulated by exercise and training, resulting in minor damage. Appropriate protein intake helps in the reconstruction and growth of muscle fibers, reduces muscle atrophy, and enhances muscle strength and endurance. Studies have shown that a diet rich in high-quality protein, such as lean meat, fish, beans, and dairy products, can significantly accelerate muscle repair and improve rehabilitation outcomes (184). Energy intake is also crucial during the rehabilitation process. Physical therapy often involves high-intensity exercise and training, which requires a large amount of energy support. Dietitians design high-energy diet plans based on the energy needs of patients to ensure they maintain sufficient energy reserves during rehabilitation, thereby reducing fatigue, enhancing endurance and physical strength, and enabling them to better participate in physical therapy (185).

**Table 5:** Essential components of nutritional intervention in the rehabilitation process.

Nutrition category	Main source	Core function
High-quality protein	Lean meat, fish, beans and dairy products	Promote muscle repair and enhance immune function (55, 184).
Carbohydrates	Whole grains, fruits	Provide energy and relieve fatigue (190).
Fat	Nuts, olive oil, deep-sea fish	Stable energy source, supporting cell functions (186).
Vitamin C and E	Citrus fruits and green leafy vegetables	Antioxidant, promoting tissue repair (196).
Vitamin D and calcium	Dairy products, fish, egg yolks	Maintain bone health and prevent bone loss (188, 196).
Zinc and magnesium	Seafood, nuts, and beans	Regulate muscle and nerve functions and enhance immunity (189).

Additionally, a reasonable intake of carbohydrates provides a sustainable energy source for patients, while fats serve as a secondary energy source, providing stable support for the long-term rehabilitation process (186). The supplementation of micronutrients (such as vitamins and minerals) also plays a vital role in the rehabilitation process. Vitamins C and E have antioxidant properties, which can reduce oxidative stress caused by intense exercise or disease, thereby promoting tissue repair (187). Vitamin D and calcium are crucial for bone health, especially for patients who have been bedridden for a long time or have limited movement, as they help maintain bone density and prevent bone loss (188). Minerals such as zinc and magnesium are involved in muscle contraction, nerve conduction, and immune function regulation (189). Therefore, during the rehabilitation process, dietitians ensure that the intake of these micronutrients is in the best balance based on the specific needs of patients (190). Physical therapy intervention, through systematic exercise and strength training, gradually restores the patient’s motor function and physical capacity. Physical therapists adjust the intensity and content of training based on the patient’s nutritional status to ensure the safety and effectiveness of the rehabilitation process. For example, for patients with poor nutritional status, physical therapists may design more moderate training programs to prevent excessive fatigue or secondary injuries caused by training. As the patient’s physical fitness improves and nutritional status enhances, physical therapists gradually increase the intensity of training to promote further recovery of muscle strength, cardiopulmonary function, and overall endurance (191). Common rehabilitation training programs include aerobic exercise, resistance training, balance training, and flexibility training, which not only help in the recovery of muscle strength but also improve cardiopulmonary endurance and overall motor ability (192, 193).

Proper nutritional support supplies essential energy and substrates for physical training, facilitating the repair and enhancement of muscles, bones, and the nervous system (194). Structured exercise regimens stimulate muscle hypertrophy, improve cardiovascular function, and augment metabolic capacity, thereby optimizing nutrient absorption and utilization (195). This reciprocal enhancement not only aids in the restoration of physiological functions in patients but also bolsters their psychological resilience and rehabilitation confidence through improved physical fitness and self-care capabilities (196). The integration of nutrition and physical therapy further elevates patients’ psychological well-being and overall quality of life. Optimal nutritional status and suitable exercise can alleviate anxiety and depression while fostering self-esteem and self-efficacy (197).

### 5.3 Integration of psychological support and clinical intervention

The profound integration of psychological support and therapeutic treatment also uncovers possible interdisciplinary synergies. This interdisciplinary therapeutic strategy efficiently facilitates patients’ physiological recovery while considerably improving their psychological adaptability and general quality of life. The diagnosis of cancer frequently induces significant psychological distress, with patients commonly encountering adverse emotions including anxiety, depression, dread, and despair (198). These emotions adversely impact patients’ quality of life and hinder their adherence to treatment and treatment outcomes. Rehabilitation psychological intervention, by thoroughly comprehending patients’ psychological states, devises individualized psychological intervention strategies, encompassing psychological counseling, emotional management skills training, relaxation techniques, and supportive group activities (199). Research indicates that these psychological interventions can markedly diminish patients’ anxiety and despair, enhance psychological resilience, and assist them in managing emotional changes and physiological problems throughout therapy (200).

In individualized psychological counseling, psychotherapists employ effective treatment modalities, such as cognitive behavioral therapy, to assist patients in reevaluating and modifying their perceptions of illness, thereby diminishing negative beliefs regarding both the disease and its treatment (111, 169). By assisting patients in identifying irrational negative thought patterns, psychotherapists can facilitate the development of more constructive coping techniques, so enabling them to sustain a more stable emotional state amongst the uncertainty of treatment plans. For example, common irrational negative thought patterns in cancer patients include catastrophizing (e.g., “I will never recover from this illness”), overgeneralization (e.g., “Since my treatment has side effects, nothing will go well for me”), and hopelessness (e.g., “There is nothing I can do to help myself”) (201). CBT helps patients recognize and challenge the validity of these distorted thoughts. Coping techniques taught in CBT include cognitive restructuring, where patients learn to replace negative thoughts with more balanced and realistic perspectives (such as, “Although treatment is challenging, there are steps I can take to improve my quality of life”), as well as behavioral activation, problem-solving skills, and relaxation techniques. By applying these strategies, CBT enables patients to reduce negative perceptions and beliefs related to their illness and treatment, thus improving psychological resilience and fostering more adaptive emotional and behavioral responses (202). The cultivation of emotional regulation abilities is a crucial element of psychological intervention. Through the instruction of relaxation techniques, deep breathing exercises, and mindfulness meditation, psychotherapists can assist patients in enhancing their self-regulation during emotional swings and preventing emotional decline (203). For example, relaxation training can diminish patients’ anxiety levels (204), but mindfulness meditation can improve their psychological focus (205), so augmenting their sense of involvement and control in therapy. These skills not only assist patients in preserving emotional stability during therapy but also markedly enhance their overall mental health, establishing a more robust psychological support base for treatment (206, 207).

Collaborative group activities can significantly contribute. In groups, patients can express their emotions and experiences, acquire understanding and support from peers, and this communal resilience can significantly mitigate individual feelings of isolation and powerlessness (208). Group activities create a secure setting for emotional expression, enabling patients to alleviate stress, cultivate strong social relationships, and enhance their confidence in the rehabilitation process (209). Research indicates that patients engaged in supportive group activities typically exhibit improved emotional regulation and life adaption, hence bolstering their psychological resilience (203). From a therapeutic treatment standpoint, the incorporation of psychological support enhances patients’ emotional well-being and augments the overall efficacy of medical interventions (210). Following psychological support, patients can more effectively comprehend each phase of the treatment plan and engage in the treatment with a more constructive disposition (211, 212). Psychotherapists can effectively collaborate with the medical team to align patients’ psychological and physiological needs within the treatment plan, so assuring holistic care for both body and mind during the treatment process (213). This integrated treatment paradigm enhances patients’ quality of life and diminishes physiological consequences resulting from psychological stress. This interdisciplinary cooperation model provides patients with rehabilitation support at the physiological level, as well as complete care at the psychological and social levels, exemplifying the fundamental principle of “holistic treatment” in contemporary rehabilitation medicine.

### 5.4 Integration of physical activity into multidisciplinary cancer care

The effective implementation of physical activity interventions for cancer patients necessitates seamless integration into a broader multidisciplinary care plan. This approach involves close collaboration among physicians (who assess medical stability and contraindications), rehabilitation experts (physical and occupational therapists who design and tailor activity plans), dietitians (who optimize nutritional support for safe exercise), pharmacists (who review medication interactions and side effects), social workers (who address psychosocial barriers), and, crucially, patients and their families (who provide feedback and support adherence) (13). Multidisciplinary team meetings can facilitate real-time adjustments to rehabilitation protocols, ensuring that physical activity is personalized and safely aligned with the patient’s evolving clinical status.

The question of whether physical activity interventions should be supervised remains a subject of ongoing debate. Current evidence suggests that supervised exercise programs, especially in the early phases of rehabilitation or for high-risk patients, are associated with improved adherence, safety, and outcomes (161). Practical models include in-clinic supervision, structured group sessions, and remote monitoring using wearable devices and telehealth platforms. For lower-risk patients or those transitioning to survivorship, hybrid or home-based programs with periodic professional oversight may be appropriate (214).

However, there remains a gap in the literature regarding standardized guidelines on supervision intensity, duration, and modality across different cancer populations and treatment stages. To address this, we propose (1) individualized risk stratification to guide the level of supervision, (2) integration of digital health monitoring tools for real-time feedback, and (3) ongoing interdisciplinary communication to promptly identify and address emerging safety concerns. Further research is warranted to establish evidence-based standards for the supervision and monitoring of physical activity in oncology rehabilitation.

## 6 Novel approaches and advanced technologies

### 6.1 Digital health and remote monitoring for cancer rehabilitation

In the realm of rehabilitation medicine, novel methodologies and advanced technology from an interdisciplinary viewpoint are continually emerging, showcasing significant potential in the prevention and rehabilitation of cancer metastasis. These technologies substantially improve the scientific rigor and customization of the intervention while also effecting transformative changes in the rehabilitation experience and quality of life for cancer patients.

In the domain of digital health and remote monitoring technologies, smart devices like smartwatches and health trackers have significantly contributed to the rehabilitation treatment of cancer patients (215). These gadgets can monitor patients’ physiological data in real time, including heart rate, activity levels, and sleep quality, while also documenting the execution of rehabilitation exercises. For individuals at elevated risk of cancer metastasis, this data can assist medical teams in recognizing physiological variations and potential health hazards (216, 217). By monitoring patients’ activity levels and weariness, the treatment team can swiftly modify the rehabilitation plan, enhance the patient’s immune function, and avert the exacerbation of inflammatory conditions, thereby diminishing the chance of metastasis (218). The utilization of remote rehabilitation platforms has transcended geographical limitations, allowing patients to perform guided rehabilitation activities at home, particularly beneficial for postoperative or physically compromised cancer patients (219). These systems, via specifically built mobile applications, offer tailored rehabilitation assistance and incorporate video call functions, enabling real-time interaction between patients and therapists (220). This ongoing and effective rehabilitation support aids cancer patients in sustaining the efficacy of systemic treatment, improving treatment adherence, and thereby diminishing the likelihood of cancer cell dissemination (221). However, limitations include variable data accuracy, technology literacy requirements for patients, and concerns regarding data privacy and security (222).

### 6.2 Virtual and augmented reality in functional and pain management

Virtual reality (VR) and augmented reality (AR) technologies have exhibited distinct advantages in the rehabilitative training of cancer patients. VR is defined as an immersive technology that completely replaces the user’s physical environment with a computer-generated, interactive digital simulation. In contrast, AR overlays digital information—such as visual graphics, spatial guidance, or auditory cues—onto the real-world environment in real-time. The fundamental difference lies in the level of environmental immersion: VR completely isolates the user from the physical world to create a newly simulated experience, whereas AR supplements the actual physical surroundings, allowing patients to remain aware of and interact with their real-world environment alongside virtual enhancements (223). By utilizing these immersive and augmented situations, patients can engage in physical therapy and exercise training within highly simulated and controlled environments (224). For cancer patients, particularly those susceptible to bone metastases or with restricted mobility post-surgery, VR technology facilitates safe and regulated movement training, significantly diminishing the likelihood of falls or injuries (225). Moreover, virtual reality technology may replicate everyday movements, such as grasping things, ambulating, or ascending and descending stairs, assisting patients in regaining fundamental functions and developing muscle memory, therefore improving their functional capabilities in real life (226, 227). In the realm of pain management, virtual reality technology diverts patients’ focus from pain via immersive activities (228). This non-pharmacological intervention is especially advantageous for the chronic pain frequently encountered by cancer patients, since it diminishes dependence on analgesics and mitigates the adverse effects of medications on the immune system, therefore reducing the chance of metastasis (229).

### 6.3 Artificial intelligence and big data for precision rehabilitation

Artificial intelligence (AI) and big data analytics significantly contribute to the management of cancer metastatic rehabilitation. Through the analysis of extensive rehabilitation data, AI technology may develop sophisticated predictive models to anticipate patient rehabilitation progress and potential metastatic concerns (230). For example, utilizing patients’ medical histories, real-time monitoring data, and genetic traits, AI models may precisely determine which patients exhibit a greater propensity for metastasis and offer tailored intervention recommendations (231). Regarding the prevention of cancer metastasis, AI can assist the treatment team in optimizing rehabilitation plans by assessing patients’ immune system status, inflammatory markers, and physical activity data, thereby rendering rehabilitation therapies more precise (230). Simultaneously, AI may adapt the intensity, frequency, and type of exercise according to patients’ rehabilitation progress, guaranteeing that their functional recovery remains within a safe range (232). Moreover, AI-driven big data analysis can amalgamate multi-dimensional patient information (including psychological state, nutritional status, and treatment adherence) to formulate more comprehensive rehabilitation strategies, thereby improving patients’ overall health and immune function while diminishing the likelihood of cancer metastasis and recurrence (233–235).

Through the integration of the aforementioned novel methodologies and advanced technologies, rehabilitation medicine has transitioned from a “experience-driven” approach to a “data-driven” paradigm in the intervention of cancer metastasis. These technologies augment the scientific rigor, accuracy, and efficacy of rehabilitation treatment, while also bolstering patients’ sense of involvement and confidence in the therapeutic process (236–238). In the future, enhanced interdisciplinary collaboration and technological integration will facilitate the advancement of personalized rehabilitation plans, offering more comprehensive metastasis prevention and functional recovery support for cancer patients, thereby improving their quality of life and long-term prognosis.

AI-guided exercise plans represent a novel paradigm in personalized cancer rehabilitation. AI algorithms can process large volumes of physiological, behavioral, and historical health data to generate real-time, individualized exercise recommendations, with consideration of patient comorbidities, treatment cycle, and symptom fluctuations. Nonetheless, translation into clinical practice is challenged by the need for validation, clinician oversight, and ethical considerations related to algorithmic transparency. Despite these promising developments, there remain significant challenges. Patient access to advanced technologies may be limited by cost or digital divides, and there is a need for rigorous clinical validation of these tools in diverse cancer populations. Additionally, integration into existing care pathways requires multidisciplinary collaboration and ongoing user education. Future research should further follow the Transparency In The reporting of Artificial INtelligence (TITAN) guidelines. These guidelines provide a standardized and comprehensive framework designed to rigorously evaluate and report the methodological design and clinical validation of AI models, thereby enhancing the transparency, reproducibility, and clinical translation of AI research reports (239).

To synthesize these advancements, **Table 6** provides a comprehensive overview of novel approaches and advanced technologies in cancer rehabilitation, highlighting specific applications and their associated clinical improvements.

**Table 6:** Summary of novel approaches and advanced technologies in cancer rehabilitation.

Advanced technology	Rehabilitation activities	Clinical statistics
AI & Big Data	Explainable AI (e.g., XGBoost combined with SHAP) for predicting mortality and recurrence risk based on multi-omics and clinical data (240).	Outperformed traditional models in predicting metastatic cancer survivability (C-index=0.70 vs. 0.66); increased precision in personalized oncology workflows (241).
Telehealth & Remote Monitoring Platforms	Supervised, personalized exercise programs delivered via telehealth (aerobic and resistance training); remote monitoring of functional tests (e.g., 30-second chair stand) (242).	Equivalent efficacy to in-person programs; significant improvements in physical function and muscular endurance; reduced perceived cognitive difficulty (243).
Nanotechnology & Wearable Sensors	Continuous, non-invasive monitoring of sweat biomarkers (e.g., cortisol, lactate) using molecularly imprinted polymer electrochemical patches (244, 245).	Enabled dynamic, real-time assessment of physiological stress and exercise intensity; high selectivity with less than 3.76% signal attenuation over long-term use (244).
VR/AR	Immersive VR distraction therapy during cancer treatments; AR-assisted spatial guidance for physical rehabilitation (246).	Reduced chronic pain intensity and procedure-related anxiety; enhanced functional mobility and patient engagement in rehabilitation protocols (247).

### 6.4 Safety considerations and risk management in exercise oncology

The implementation of exercise interventions in cancer rehabilitation must be carefully balanced with safety considerations, given the unique risks associated with oncologic populations (248). Exercise is generally recognized as safe and beneficial for most cancer patients; however, several contraindications and risk factors should be considered (249). Absolute contraindications to exercise include severe anemia, acute infections, uncontrolled pain, significant cardiopulmonary compromise, and the presence of unstable bone lesions that may predispose patients to pathological fractures, particularly in those with bone metastases. Relative contraindications, such as thrombocytopenia or moderate anemia, require individualized assessment and modified exercise intensity (250).

Risk management strategies involve pre-exercise screening by a multidisciplinary team, ongoing symptom monitoring, and immediate cessation of exercise in the presence of warning signs such as chest pain, sudden dyspnea, dizziness, or new bone pain (251). Exercise prescriptions should be tailored according to each patient’s clinical status, treatment phase, and functional capacity, with close supervision for high-risk individuals (252). The integration of digital health monitoring and remote supervision can further enhance safety by enabling real-time assessment and rapid response to emergent issues (253). Continuous staff education and patient counseling are critical to ensure both safety and adherence throughout the rehabilitation process (254).

## 7 Challenges and future prospects of rehabilitation interventions

### 7.1 Challenges of individual variability and psychological factors

Individual differences present considerable problems in the prevention and recovery of cancer metastases. Each cancer patient possesses distinct physiological traits, genetic profiles, and immunological responses, resulting in the same therapy and rehabilitation strategies yielding significantly varied outcomes among patients (255). For example, during chemotherapy, radiation, or targeted therapy, several patients may demonstrate entirely distinct side effects or therapeutic results. This not only impedes the seamless advancement of treatment but may also modify the dynamics of metastatic risk (256). Consequently, developing individualized rehabilitation strategies for cancer metastasis necessitates a thorough evaluation of each patient’s distinct physiological characteristics, pathological mechanisms, and therapy responses (257, 258). To realize this comprehensive evaluation, a variety of assessment tools and procedures are utilized. These include gene mapping or genetic profiling to identify mutations or genetic variants that may impact disease progression and treatment response. Biomarkers such as circulating tumor cells, circulating free DNA, and protein markers are regularly monitored to assess disease status and therapeutic efficacy (259, 260). In addition, patients undergo regular clinical follow-up visits and imaging studies, allowing for dynamic monitoring of the treatment course. Continuous assessment through laboratory tests, symptom evaluation, and functional status measurements ensures timely adjustment of rehabilitation strategies and lends authenticity and reliability to the evaluation process (261, 262). The psychological state, emotional variations, and attitudes towards the disease and treatment acceptance of patients considerably affect the efficacy of rehabilitation programs (263, 264). Emotional anxiety, despair, and treatment resistance can impair immunological function, which is a significant factor in the proliferation and spreading of cancer cells (265, 266). Consequently, rehabilitation strategies for cancer metastasis must encompass not only physiological treatment but also extensive psychological support and emotional management to attain multifaceted intervention objectives (267).

### 7.2 Continuity in rehabilitation and the role of emerging technologies

The continuity and consistency throughout the rehabilitation process present a considerable difficulty in preventing cancer spread. Cancer patients may disrupt their rehabilitation programs due to medication side effects, physical weariness, or diminished motivation, therefore heightening the risk of cancer spreading during periods of instability or interruption in the rehabilitation process (268). Consequently, the rehabilitation team must exhibit significant flexibility and reactivity, enabling prompt adjustments to the rehabilitation plan based on the patient’s treatment progress and specific circumstances (269). For patients undergoing chemotherapy, the rehabilitation plan may necessitate a reduction in exercise intensity to mitigate physical strain, while addressing immune function decline through nutritional support and psychological intervention, thereby diminishing the likelihood of cancer cell dissemination (270).

Technological progress has introduced novel methods for the prevention and rehabilitation of cancer metastasis, although it also presents significant obstacles (271). Advanced technologies, including artificial intelligence, big data analytics, and nanotechnology, provide a scientific foundation for individualized rehabilitation (272, 273). Specifically, nanotechnology assists in individualized rehabilitation through the deployment of advanced wearable and implantable nanosensors (274). These nanoscale diagnostic tools enable the continuous, non-invasive monitoring of critical physiological biomarkers, such as metabolic fluctuations, inflammatory cytokines, and oxidative stress levels directly from sweat or interstitial fluid during exercise (275). By capturing these real-time molecular insights, rehabilitation teams can precisely tailor the intensity and duration of physical therapies to a patient’s immediate physiological tolerance, thereby optimizing recovery while minimizing the risk of exercise-induced systemic stress (276). Through the real-time analysis of patients’ physiological data and illness progression, AI technology can assist the treatment team in predicting the likelihood of cancer spreading and devising extremely precise intervention strategies (277). For example, several studies have reported that the implementation of AI–assisted clinical decision support systems can improve the accuracy of cancer-related risk predictions by 15–20% compared to conventional methods (278, 279). Moreover, data-driven and AI-supported rehabilitation management has been shown to enhance patient adherence and the overall effectiveness of individualized rehabilitation interventions (280). These findings underscore the tangible impact of advanced technologies on both the assessment and management of patients with metastatic cancer. Utilizing these forecasts, the rehabilitation team can formulate preemptive anti-inflammatory and immune-boosting therapies. Nonetheless, the implementation of these novel technologies requires validation through rigorous scientific research to ascertain their efficacy, safety, and broad applicability. The expenses and intricacies associated with emerging technologies may impose greater demands on patients, medical institutions, and rehabilitation teams (281). Achieving technological accessibility and universality with constrained resources is a pressing issue that requires resolution (282).

### 7.3 The importance of patient education and social support systems

Patient education and social support are essential components in the recovery phase of cancer metastasis (283, 284). Patients and their families may possess insufficient understanding of tumor metastatic mechanisms and rehabilitation treatments, resulting in a failure to comprehend the necessity of rehabilitation interventions and the significance of sustained commitment (285). Consequently, rigorous patient education is essential, particularly in disseminating knowledge about the risk factors associated with cancer metastasis (including chronic inflammation and lifestyle) and rehabilitation techniques, to enable patients to engage actively in their rehabilitation process (286, 287). Cancer patients may encounter adverse feelings, including loneliness, anxiety, and sadness throughout rehabilitation, which may further compromise immune function and elevate the chance of metastasis (288, 289). Consequently, establishing a robust social support network and psychological intervention system for patients might augment their rehabilitation motivation, bolster their confidence in life, and thereby indirectly diminish the likelihood of cancer spreading (290, 291).

Individual characteristics, psychological variables, and the interplay between technology and social support are essential components that must be considered in the rehabilitation intervention for cancer metastases (292). Enhancing interdisciplinary collaboration, refining personalized rehabilitation approaches, and advancing technological innovations can effectively tackle various challenges in cancer metastasis prevention, offering comprehensive support for patients and ultimately enhancing their long-term prognosis and quality of life (293).

To further strengthen the interdisciplinary framework of metastasis rehabilitation, integrated treatment models involving multidisciplinary teams (MDTs) have become standard practice in many comprehensive cancer centers (294). Typical MDTs comprise oncologists, rehabilitation physicians, physiotherapists, occupational therapists, psychologists, nutritionists, and social workers. These teams work collaboratively to design and implement individualized rehabilitation plans tailored to the complex needs of patients at risk for or experiencing metastasis (295).

One commonly employed integrated model is the MDT case conference approach, in which regular meetings are held to discuss each patient’s oncologic status, functional assessment, psychosocial needs, and progress in rehabilitation (296). For example, a patient with lung cancer and bone metastases may be presented at a weekly MDT meeting: the oncologist discusses current tumor management, the rehabilitation specialist evaluates physical limitations and risk of complications, while the physiotherapist and occupational therapist develop a tailored exercise and adaptation plan. The psychologist addresses emotional needs, and the nutritionist adjusts dietary recommendations to support systemic treatment and rehabilitation. This collaborative and dynamic approach allows the team to promptly modify interventions according to changes in the patient’s condition, treatment side effects, and personal goals (297).

Case studies have illustrated the effectiveness of these models. For instance, a published case from a tertiary cancer center detailed how an MDT developed a comprehensive rehabilitation protocol for a breast cancer patient with spinal metastasis. Through coordinated interventions, including pain management, mobility training, adaptive equipment provision, and ongoing psychosocial support, the patient achieved improved functional independence and quality of life, with better adherence to both oncologic and rehabilitation treatments (298, 299).

Moreover, the integration of digital health platforms into MDT workflows enables remote monitoring and real-time communication among team members and with patients, further enhancing continuity of care. These team-based, patient-centered approaches have been shown to improve functional outcomes, reduce complications, and increase patient satisfaction in cancer metastasis rehabilitation (300, 301).

### 7.4 Balanced view and current evidence limitations

While numerous studies have highlighted the potential benefits of rehabilitation interventions in improving quality of life and functional status in cancer patients, the evidence on their direct effect in preventing or reducing cancer metastasis remains inconclusive. Several systematic reviews and clinical studies report that, although exercise and other rehabilitation approaches can enhance physical function and psychological well-being, their direct impact on metastasis-free survival or delayed metastatic progression is less clear (150, 302). For example, some randomized controlled trials have not demonstrated a statistically significant difference in metastatic outcomes between patients receiving rehabilitation interventions and those receiving standard care. These neutral or inconclusive findings may be attributed to methodological shortcomings, primarily small sample sizes and the profound heterogeneity of exercise protocols. Variations in exercise modality, intensity, frequency, and duration across different studies make cross-study comparisons and the establishment of standardized clinical guidelines difficult (303).

Furthermore, a significant gap exists between preclinical successes and clinical realities. While animal models have provided robust mechanistic insights into how exercise or rehabilitation modifies the TME and immune responses, translating these *in vivo* findings into human clinical practice remains highly challenging. Animal models often fail to replicate the complex genomic heterogeneity of human tumors, the compounding effects of concurrent systemic therapies, and the prolonged, multifactorial clinical course experienced by cancer patients.

Moreover, certain high-risk patient groups—such as those with advanced disease, bone metastases, or severe comorbidities—may not experience the same benefits from rehabilitation interventions, and in some cases, could be at risk of adverse events if interventions are not properly individualized and closely monitored (304). This underscores the necessity for robust pre-intervention risk assessments and personalized rehabilitation plans that take into account the patient’s unique clinical status, cancer stage, and treatment goals.

It is also important to note that most current studies primarily focus on surrogate or secondary endpoints (such as physical function, fatigue, or quality of life) rather than metastasis or overall survival as primary outcomes. Crucially, there is a distinct lack of long-term metastatic outcome data. Most clinical trials feature relatively short follow-up periods, which are insufficient to capture latent metastatic events. The lack of high-quality, long-term trials specifically addressing metastatic progression limits our ability to draw definitive conclusions about the anti-metastatic effect of rehabilitation (243). Therefore, rehabilitation interventions should not be viewed as universally suitable or beneficial for all cancer patients across all disease stages. The design and implementation of rehabilitation strategies must be carefully tailored and supported by multidisciplinary teams, with continuous re-evaluation and monitoring to ensure both safety and efficacy.

Further high-quality, large-scale, and long-term studies are required to clarify the precise role of rehabilitation in metastasis prevention and management, and to identify which patient subgroups are most likely to benefit (305). Until then, a cautious and individualized approach is warranted, balancing the potential benefits of rehabilitation against the possible risks in vulnerable populations.

Despite these limitations, the synthesis of current evidence carries profound overall clinical implications, advocating for a paradigm shift from traditional, reactive rehabilitation to proactive, precision-based oncology rehabilitation. Integrating tailored exercise, physical modalities, and psychosocial support into the standard continuum of multidisciplinary cancer care can optimize the systemic neuroendocrine-immune network and remodel the local TME, creating a less permissive state for metastatic colonization. To bridge the existing translational gap between preclinical success and clinical reality, future research must prioritize several strategic directions. First, future RCTs must pivot from relying solely on secondary quality-of-life metrics to prioritizing metastasis-free survival and overall survival as primary endpoints. Second, research should integrate liquid biopsies and multi-omics profiling to continuously monitor metastatic risk, enabling the formulation of precise “exercise dosing” tailored to a patient’s real-time immune and inflammatory biomarker fluctuations. Third, deeper investigations are required to map the exact molecular docking mechanisms and intracellular signaling cascades triggered by specific physical modalities. Furthermore, clinical trials must be designed with extended follow-up periods exceeding one year to accurately capture latent metastatic events and evaluate the sustained efficacy of long-term rehabilitation adherence. Finally, future studies should utilize digital health platforms, wearable nanosensors, and AI-driven analytics to harmonize intervention protocols, thereby ensuring reproducibility across diverse global cohorts.

## 8 Conclusion

This review examines the multifaceted function of rehabilitation interventions in avoiding cancer metastasis, emphasizing the need of interdisciplinary collaboration in this domain. By synthesizing expertise from several fields including clinical medicine, exercise therapy, physical modality therapy, occupational therapy, and psychotherapy, tailored and holistic treatment strategies can be devised for patients, significantly mitigating the risk of cancer spread. Nonetheless, rehabilitation interventions for cancer metastasis encounter numerous hurdles, including individual variability, psychosocial conditions, and technological implementation. Each patient possesses distinct physiological traits, genetic origins, and immunological responses, necessitating the development of tailored intervention programs. Simultaneously, emphasis must be placed on psychological support and emotional management to mitigate the risk of metastasis. Moreover, technical advancements like artificial intelligence, big data analytics, and nanotechnology present novel opportunities for rehabilitation interventions; yet, their implementation necessitates proof of efficacy and resolution of accessibility challenges. Enhancing patient education and social support will facilitate improved rehabilitation adherence and mental well-being, thereby diminishing the likelihood of cancer cell metastasis.

Nevertheless, it should be acknowledged that patient responses to rehabilitation interventions are highly heterogeneous due to variations in tumor type, disease stage, and individual physical condition, which may affect generalizability. Additionally, logistical barriers such as limited access to specialized services and disparities in the availability of technological resources can restrict the implementation of these interventions in real-world settings. The increasing use of technology and digital tools in rehabilitation also raises ethical concerns regarding data privacy and equitable access, which warrant careful consideration in future applications and research. Future research should investigate the differential impacts of specific rehabilitation approaches on metastasis inhibition across various cancer types to elucidate optimal circumstances and strategies for rehabilitation intervention. Simultaneously, long-term follow-up studies are crucial for assessing the enduring effects of rehabilitative interventions, as they evaluate short-term efficacy and offer a scientific foundation for the long-term prevention of cancer recurrence and metastasis. Furthermore, comprehensive investigation into the molecular mechanisms underlying rehabilitation interventions, including their modulation of cytokines, immune cells, and matrix constituents within the TME, will furnish theoretical foundations for the formulation of more targeted rehabilitation strategies. These initiatives will facilitate the shift of rehabilitation medicine from conventional empirical approaches to mechanism-based precision medicine, thereby offering enhanced rehabilitation care for cancer patients and ultimately enhancing their long-term prognosis and quality of life.
